# Filling the void: sense of absence as a comprehensive bridge between attachment, parent–adolescent communication, and addictive behaviors among adolescents

**DOI:** 10.1186/s13034-026-01150-0

**Published:** 2026-08-05

**Authors:** Yaniv Efrati

**Affiliations:** Faculty of Education, Bar-Ilan University, Ramat Gan, Israel

**Keywords:** Sense of absence, Addictive behaviors, Attachment, parent-adolescent communication, Network analysis

## Abstract

Adolescent addictive behaviors present a growing public health concern, yet the pathways leading from experiences of disconnection to persistent addictive patterns remain unclear. This study examined the Sense of Absence (SoA), a subjective feeling of inner void and disconnection, as a potential bridge linking relational deprivation with addictive behaviors. This cross-sectional study used network analysis and was conducted in Israel with a sample of 2,564 adolescents’ communities. Participants completed a sense of absence, relational tendencies (parent-adolescent communication, attachment avoidance, attachment anxiety) and addictive behaviors (alcohol, drugs, internet gaming disorder, problematic social networks use, smartphone addiction, shopping addiction, problem gambling, problematic pornography use, compulsive sexual behavior). Bridge centrality analysis indicated that SoA exhibited the highest bridge strength across all nodes, exceeding direct relational to addiction connections. Connection density analysis showed that SoA had the strongest cross domain connectivity, whereas direct edges between relational and addictive domains were relatively sparse. SoA also showed stronger associations with relational factors than with addictive behaviors. Overall, the findings suggest that SoA occupies a central structural position in the network linking relational tendencies and addictive behaviors.

**Supplementary Information:**

The online version contains supplementary material available at https://doi.org/10.1186/s13034-026-01150-0.

## Introduction

A substantial body of work in attachment theory and affective neuroscience indicates that addictive behaviors emerge not only from the search for pleasure but also from attempts to soothe an inner sense of emptiness and ongoing disconnection from oneself and from others. Early disturbances in attachment can alter the functioning of dopamine, opioid, and oxytocin systems, pushing the developing brain toward stress driven habit circuits while weakening capacities for social learning and secure bonding. In this context, both pharmacological and behavioral rewards become especially appealing because they offer brief relief from feelings of absence and detachment [1–3]. Research in social identity further shows that experiences of isolation and a diminished sense of belonging often precedes substance involvement, with drug using peer groups providing temporary sources of identity and connection [4]. Findings on anhedonia support this pattern as well; individuals who are vulnerable tend to exhibit weaker responses to natural rewards, while substances and certain online addictive behaviors artificially stimulate mesolimbic and midbrain pathways, offering short term regulation of emotional emptiness and disconnection [5]. These mechanisms are particularly salient in adolescence, a developmental stage marked by significant changes in social reward sensitivity, during which stress from social exclusion can heighten later vulnerability to opioid seeking behavior [6].

## Sense of absence

Against this backdrop, the SoA construct was recently proposed to capture experiences of inner missingness, relational invisibility, and difficulty engaging fully in life [7]. The idea of absence has occupied both philosophy and psychology for many years, with scholars debating whether it reflects perceptual, cognitive, or metacognitive processes that register what is no longer present [8]. Developmental and psychoanalytic perspectives have similarly emphasized that early experiences of unavailability shape the emerging self. Freud [9] highlighted the mind’s efforts to defend against the pain of loss, while Winnicott [10], Fairbairn [11], and later Bowlby [12] showed how inconsistent or unresponsive caregiving can leave children with enduring feelings of emptiness, vigilance, or relational avoidance. These early adaptations may continue into adolescence and adulthood, shaping emotional regulation and patterns of connection [13, 14].

From a developmental standpoint, the SoA reflects a subjective state marked by relational invisibility and inner disconnection. While traditional measures of emptiness emphasize internal hollowness and meaninglessness, this construct draws attention to the experience of being present yet unnoticed, consistent with recent work on anti-mattering as a distinct interpersonal risk factor [15]. It also highlights a form of existential detachment in which adolescents feel as if they are observing life rather than participating in it, a difficulty that differs from anhedonia because it concerns the capacity to engage rather than the ability to feel pleasure [5]. In addition, the SoA captures disruptions in self-continuity and vitality, often expressed through a struggle to feel love or to extend it to others, a pattern that reaches beyond the passive emotional pain described in psychache models [16]. Taken together, this construct represents a form of affective and relational distress with a distinct existential quality. This profile may help explain why some individuals turn to addictive behaviors as a compensatory strategy, aligning with self medication approaches that link substance use to attempts at regulating painful internal states [17].

This distinction may be clarified further by considering SoA alongside anti-mattering and loneliness in the context of addictive behaviors [18–20]. Although these constructs overlap in their emphasis on disconnection and diminished social presence, they refer to different levels of subjective experience. Anti-mattering primarily reflects the interpersonal appraisal that one is insignificant, unnoticed, or of little consequence to others [21]. Loneliness, in contrast, refers to the distressing emotional experience of perceived isolation or insufficient social connection [22]. SoA extends beyond both constructs by capturing a broader existential and relational condition in which the adolescent experiences not only social disconnection, but also inner emptiness, diminished vitality, and reduced engagement with life and relationships [7].

In this sense, anti-mattering concerns perceived interpersonal insignificance, loneliness concerns the emotional pain of disconnection, and SoA reflects a more pervasive subjective state of internal absence. This distinction is especially relevant for addiction research, because adolescents may engage in addictive behaviors not only to cope with rejection or isolation, but also to compensate for a deeper sense of inner emptiness and disengagement. From this perspective, addictive behaviors may serve as maladaptive attempts to generate temporary stimulation, relief, or a sense of subjective presence.

## Parent–adolescent communication

Family systems theory views parent adolescent communication as a key mechanism through which the emotional climate of the home is shaped and regulated [23]. Research shows that parental understanding depends largely on adolescents’ willingness to share, which emerges in relationships grounded in trust and reciprocity rather than in parental control [24]. In such climates, distress can be expressed and contained. When communication is marked by criticism or emotional distance, adolescents may withdraw, leaving the SoA unspoken and more likely to intensify. Under these conditions, feelings of emptiness can be managed through addictive behaviors that offer temporary relief. Empirical evidence supports this view, showing that warm and open communication is associated with lower involvement in addictive behaviors [25–28].

## Attachment orientation

Attachment style shapes how adolescents experience and respond to SoA. Secure attachment, marked by trust and comfort with seeking support, promotes effective regulation of distress and allows feelings of absence to be acknowledged and worked through rather than immediately avoided [29, 30]. In contrast, insecure attachment can intensify this experience. Adolescents with anxious attachment often interpret absence as rejection and may seek relief through compulsive attempts to reconnect or through emotional numbing [31, 32]. Those with avoidant tendencies may suppress distress until it is expressed through behaviors that offer control without intimacy, such as substance use or online behaviors [2, 33, 34]. Through these processes, the SoA becomes an enduring relational state that contributes to the development of addictive behaviors by shaping affect regulation and coping. It is important to note that this construct is not simply another marker of attachment insecurity. Attachment reflects broad expectations about oneself and others, while the SoA captures the immediate, subjective feeling of being unseen and unable to participate in shared experience. This distinction aligns with neurobiological evidence that addiction and insecure bonding involve dopaminergic and oxytocin related processes, while the lived experience of relational absence adds another layer of explanation for why adolescents may seek substances or online activities as substitutes for connection and engagement [3].

Taken together, these perspectives suggest a relational-developmental account of addictive engagement. When attachment relationships and parent–adolescent communication do not provide reliable recognition, co-regulation, and emotional soothing, adolescents may have greater difficulty regulating distress and maintaining a stable sense of connection. The SoA may represent the subjective expression of this relational deficit: not merely the absence of another person, but the felt absence of secure regulation, internalized support, vitality, and meaningful participation. Under such conditions, substances and potentially addictive behaviors may become attractive as external regulators because they can temporarily numb distress, provide stimulation, narrow attention, or simulate connection. With repetition and reinforcement, this compensatory use may become increasingly habitual or compulsive, particularly when emotion-regulation difficulties are also present. This formulation integrates attachment-based and developmental models of addiction with compensatory-use and transdiagnostic models, positioning SoA as a possible experiential link between relational difficulties and diverse addictive behaviors, while recognizing that the evidence is strongest for substance use and problematic internet- or media-related behaviors [29, 32, 35–38].

## In the current study

The present study, extending the work of Efrati and Pontaza [7], explored how the sense of absence functions within adolescents’ psychological networks and how it connects relational tendencies such as parent adolescent-communication, attachment avoidance, and attachment anxiety with various addictive behaviors. By conceptualizing the sense of absence as a lived experience of inner void, disconnection, and reduced emotional presence, the study builds on evidence that feelings of emptiness often accompany addiction. This perspective conceptualizes addiction as associated with experiences of absence. Within this framework, the study examined whether SoA occupies a central position linking relational tendencies and addictive behaviors.

## Method

### Participants

The sample consisted of 2,564 adolescents from the general community in Israel. Participants had a mean age of 16.41 years (SD = 1.48). The sample included 59.3% females, 40.4% males, and 0.2% participants with unknown sex data. Adolescents were enrolled across middle and high school grades, including 8th grade (5.4%), 9th grade (11%), 10th grade (21%), 11th grade (25%), and 12th grade (37%), with grade information missing for 61 participants. The vast majority of participants were born in Israel (97%), while 3% were born elsewhere. Regarding religiosity, 955 (37.3%) identified as secular, 816 (31.8%) as traditional, 710 (27.7%) as religious, 37 (1.4%) as ultra-Orthodox, and 46 (1.8%) as other. Most participants reported good (63%) or very good (30%) family economic status, with a small proportion reporting bad (5.1%) or very bad (1.3%) economic conditions.

### Procedure

Participants were informed that the study examined emotional and psychological experiences among Israeli adolescents from a wide range of backgrounds, including sex, levels of religiosity, and different regions of Israel. All measures were administered online through the Qualtrics platform with the assistance of trained research staff. Parents of interested adolescents received detailed information by phone or email and were asked to provide electronic written consent before their children could take part. After parental consent was obtained, adolescents received an anonymous survey link. The questionnaires were presented in a randomized order to limit response bias. After completing the survey, participants received an online debriefing and were thanked for their participation.

The study was conducted in accordance with the ethical principles of the Declaration of Helsinki and was approved by the Institutional Ethics Committee of Bar-Ilan University (No. 310825719). All methods were carried out in accordance with relevant guidelines and regulations. Electronic written informed consent was obtained from parents prior to adolescents’ participation, and informed assent was obtained from the adolescent participants.

### Measures

The questionnaires used in the current study are presented in Table 1.

**Table 1:** Questionnaires used in the current study

Characteristic	Questionnaire to be used	Brief description	Example of an item	Reliability alpha	Reliability omega
Sense of absence	Sense of Absence Scale (SoAS; [7])	14 items measuring experience of inner void, emotional disconnection, and diminished sense of presence	I feel alone in the world, as if no one understands me	0.96	0.96
Attachment style	Experiences in Close Relationships Scale (ECR; [39])	36 items that measure the two main dimensions of adult attachment styles – anxious and avoidant attachment	Feel close to others	Anxiety 0.89 Avoidance 0.85	Anxiety 0.91 Avoidance 0.89
Parent- Adolescent communication	Parent-Adolescent Communication Scale (PACS; [40])	20-item tool designed to evaluate the quality of communication between adolescents and their parents	My mother/father is always a good listener	0.91	0.93
Alcohol	Alcohol Use Disorders Identification Test (AUDIT; [41])	10 items measuring alcohol addiction symptoms	During the past year, how often have you felt guilt or remorse after drinking?	0.91	0.93
Drugs	Drug Abuse Screening Test (DAST-10; [42])	10 items measuring drug addiction symptoms experienced during the past 12 months: yes/no response (higher scores=more severity)	Are you unable to stop abusing drugs when you want to?	0.81	0.86
Gaming	Internet Gaming Disorder (IGDS9-SF; [43])	9 items that evaluate the severity of IGD and its adverse effects over a 12-month period	Do you feel more irritability, anxiety or even sadness when you try to either reduce or stop your gaming activity?	0.95	0.96
Social Networks	Problematic Social Networks Use (PSNU; [44])	9 items that evaluate the severity of PSNU and its adverse effects over a 12-month period	Do you regularly find that you can’t think of anything else but the moment that you will be able to use social media again?	0.93	0.95
Smartphone	Problematic smartphone use (short version SAS -SV; [45])	10-item smartphone addiction scale (SAS-short version) was used to assess the extent of smartphone addiction	missing planned work due to smartphone use	0.93	0.95
Shopping	Bergen Shopping Addiction Scale (BSAS; [46])	7 items measuring shopping addiction symptoms experienced during the past 12 months	I shop/buy things in order to change my mood.	0.89	0.92
Gambling	Problem Gambling Severity Index (PGSI; [47])	9 items that evaluate the severity of PG and its adverse effects over a 12-month period	Have you felt that you might have a problem with gambling?	0.95	0.96
Pornography	Brief Pornography Screen (BPS; [48])	4 items that evaluate the severity of pornography and its adverse effects over a 6-month period	You find it difficult to resist strong urges to use pornography.	0.92	0.93
Sex	Compulsive Sexual Behavior Disorder Scale (CSBD-7; [49])	7 items that comprehensively assess compulsive sexual urges, thoughts, and behaviors and their consequences in the past 6 months	My sexual desires control me.	0.92	0.93

### Data analysis

The analyses included all 2,564 participants. Across the 13 composite variables entered into the network analysis, 12.72% of the 33,332 possible participant-by-variable cells were missing. Thus, the reported percentage represents cell-wise missingness rather than the proportion of participants with one or more missing values. The analytic dataset contained 101 distinct case-level patterns of missingness across these 13 variables. Missing values for descriptive demographic variables, including grade information for 61 participants and sex information for 6 participants, were reported separately and were not included in the 12.72% calculation or in the multiple-imputation model. Before the primary analyses, we identified the type of missing data using Jamshidian and Jalal’s non-parametric Missing Completely At Random (MCAR) test. We found that the data were not Missing Completely At Random, Hawkins’s χ²median (36) = 290.23, *p* < 0.01, Anderson-Darling’s Tmedian = 47.54, pmedian < 0.01. The significant results from both tests indicated rejection of the MCAR assumption, suggesting the data were Missing At Random (MAR). Accordingly, missing data were handled using multiple imputation with the mice and micemd R packages. Fourteen completed datasets were generated using parallel processing (*m* = 14, nnodes = 10, maxit = 10). The number of imputations was selected to slightly exceed the overall cell-wise missingness rate of 12.72% and to reduce Monte Carlo variability.

Additionally, outlier detection was conducted using robust Minimum Volume Ellipsoid (MVE) estimation. Robust Mahalanobis distances based on MVE identified 585 cases exceeding the 97.5% χ² cutoff. These cases were retained in the analyses and neither removed nor downweighted during network estimation. The outlier assessment was used diagnostically, while regularized network estimation and bootstrap procedures were used to evaluate the robustness and stability of the findings. The Anderson-Darling test revealed a significant departure from multivariate normality (minimal A = 2.18, *p* < 0.01), confirming that all variables were non-normally distributed. To address this non-normality, bootstrap procedures were implemented in subsequent network analyses to ensure robust statistical inference and confidence interval estimation.

Then, the network structure was estimated using the Extended Bayesian Information Criterion Graphical Least Absolute Shrinkage and Selection Operator (EBICglasso). Correlations were selected according to variable type, including polychoric correlations between ordinal variables, polyserial correlations between ordinal and continuous variables, and Pearson correlations between continuous variables. Network estimation was conducted with the estimateNetwork function in the bootnet package. The procedure searched across 1,000 regularization parameters to identify the network that provided the optimal balance between goodness of fit and parsimony. The network was subsequently refitted, and statistical thresholding was applied to reduce the retention of potentially spurious edges.

The resulting network was examined to describe the conditional associations among Sense of Absence, relational variables, and addictive behaviors. Overall node centrality was evaluated using four complementary indices: (i) strength, defined as the sum of the absolute weights of all edges connected to a node; (ii) closeness, reflecting the average distance between a node and all other nodes in the network; (iii) betweenness, representing the frequency with which a node lies on the shortest paths between other nodes; and (iv) expected influence, defined as the sum of the signed edge weights connected to a node and therefore retaining both positive and negative associations.

Before calculating bridge centrality, community membership was empirically examined using bootstrap exploratory graph analysis (bootEGA) from the EGAnet package. The analysis was conducted on the same 13 nodes included in the network and used GLASSO network estimation, the Walktrap community-detection algorithm, and 5,000 nonparametric case-resampling iterations. The frequency with which alternative community solutions were recovered, the structural consistency of the identified communities, and the stability of individual node assignments were examined. The empirically derived community memberships were then used in the bridge-centrality analysis, thereby avoiding the a priori designation of Sense of Absence as a separate singleton bridge category.

Conventional bridge-centrality indices were calculated using the bridge function from the networktools package. One-step bridge expected influence was defined as the sum of the signed edge weights connecting a node to nodes outside its empirically assigned community. Two-step bridge expected influence additionally incorporated indirect cross-community connectivity through adjacent nodes. Bridge strength was defined as the sum of the absolute weights of all edges connecting a node to nodes in other communities. Because the estimated network included both positive and negative edges, one-step and two-step bridge expected influence were treated as the primary bridge indices, whereas bridge strength was interpreted as a secondary measure because it does not retain edge direction. Bridge closeness and bridge betweenness were not interpreted because their calculation requires the removal of negative edges. Normalized bridge indices were used for primary comparisons to account for differences in the number of possible cross-community connections across communities.

Network stability was evaluated using a case-dropping bootstrap with 5,000 iterations. Correlation-stability coefficients were calculated for strength, expected influence, closeness, and betweenness to assess the extent to which centrality estimates remained consistent after progressively larger proportions of the sample were removed. All analyses were conducted in R using the bootnet, qgraph, EGAnet, and networktools packages.

## Results

### Network structure and centrality analysis

**Fig. 1:**
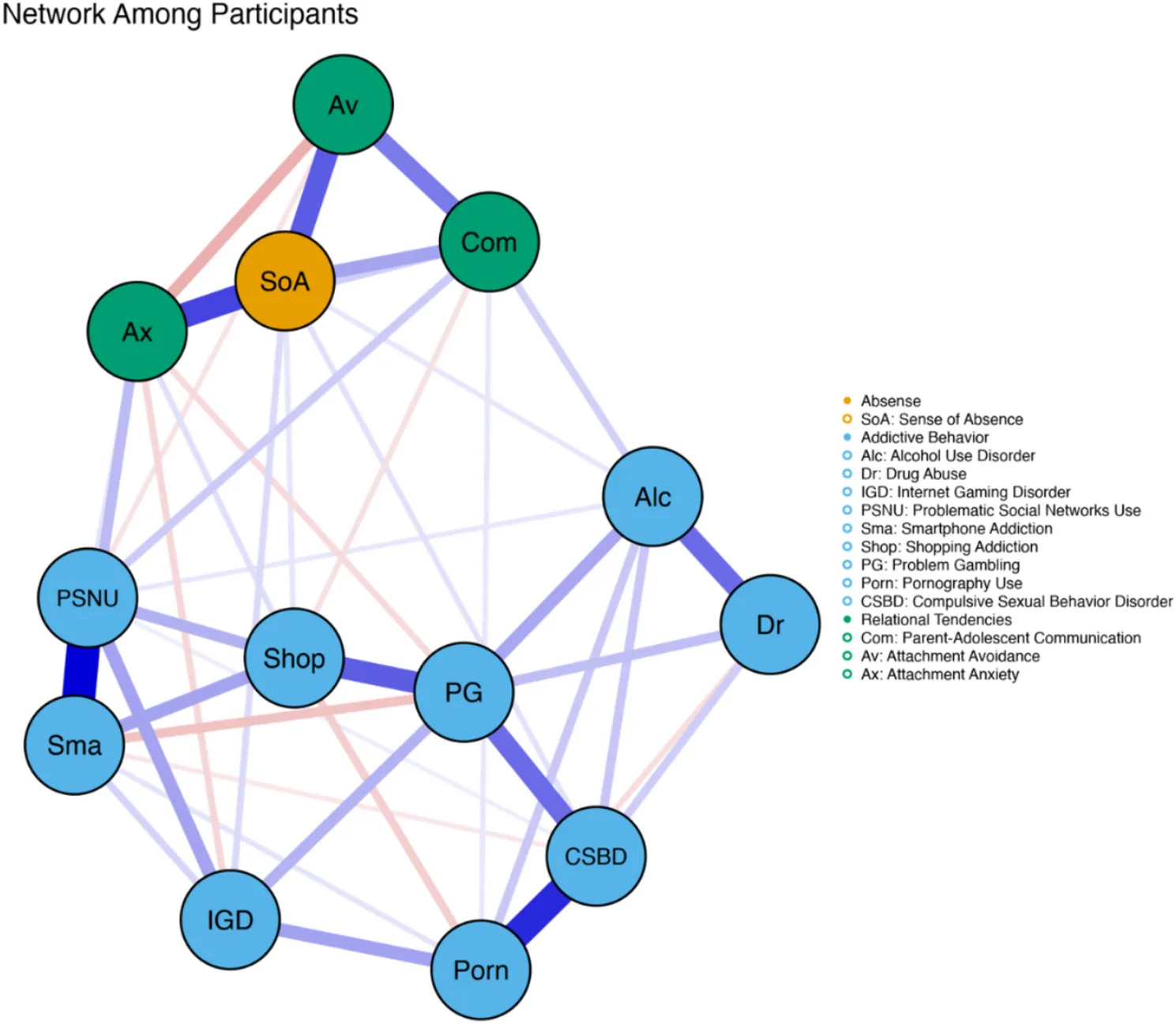
Network analysis of addictive behaviors measures, relational tendencies, and sense of absence. Blue edges (i.e., paths) reflect positive associations, whereas red edges reflect negative associations. The edges’ brightness, distance, and width reflect different relative strength indicators. Nodes’ colors highlight the domain: relational tendencies (green), addictive behaviors (light blue), and sense of absence (orange)

Figure 1 presents the network structure among sense of absence, relational tendencies, and addictive behaviors (*N* = 2,564). The network analysis revealed a densely connected structure with 13 nodes representing the key psychological and behavioral variables. Visual inspection showed sense of absence (SoA) positioned centrally within the network, with connections spanning both relational and addictive behavioral domains.

**Fig. 2:**
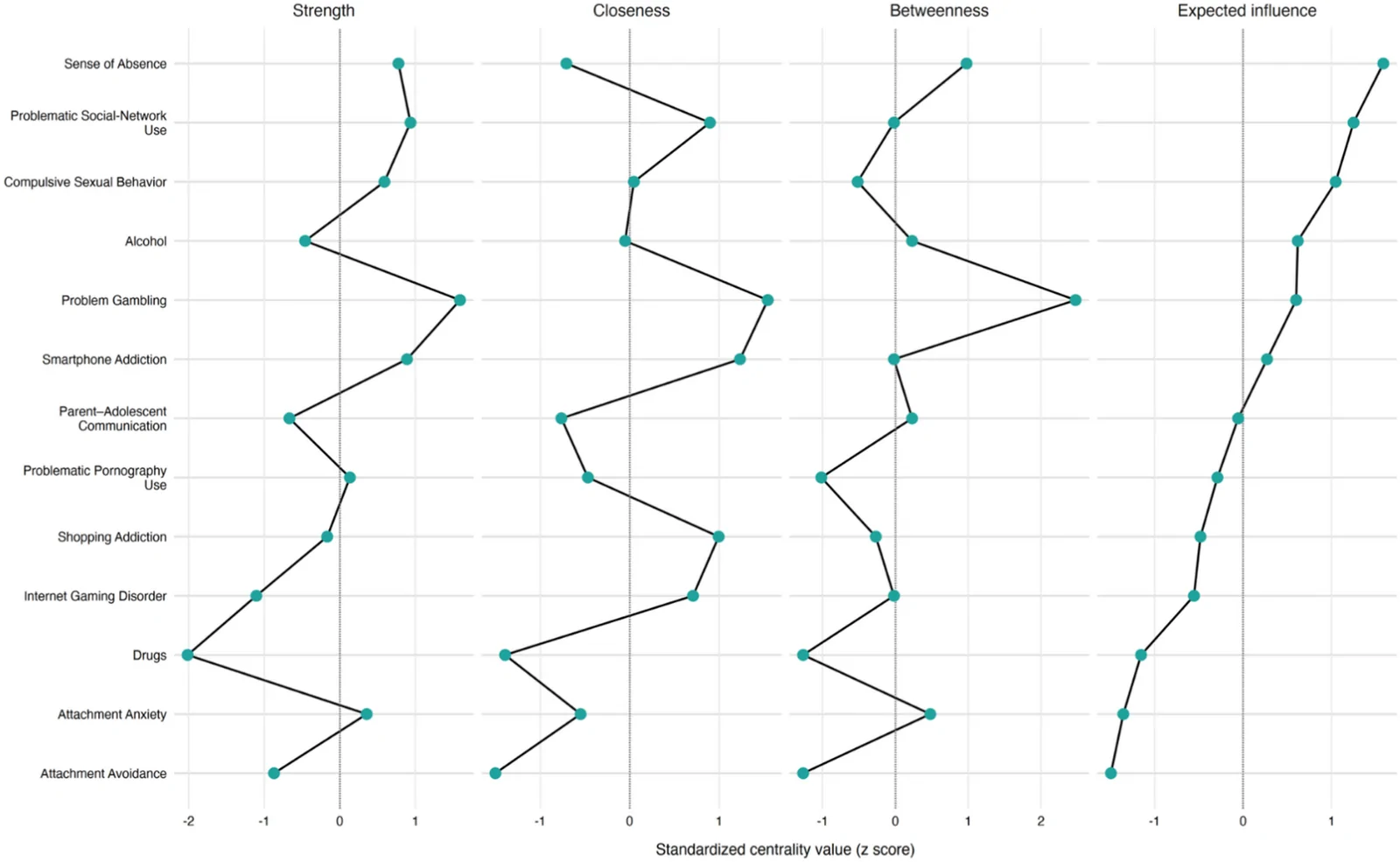
Standardized centrality indices for the estimated network. Nodes are ordered according to expected influence. Centrality values are presented as standardized *z* scores within each index, such that positive values indicate above-average centrality and negative values indicate below-average centrality relative to the other nodes in the network. The x-axis range is allowed to vary across panels to improve readability. This ordering applies only to expected influence and should not be interpreted as indicating that Sense of Absence ranked highest across all centrality measures. Sense of Absence ranked first in expected influence, second in betweenness, fourth in strength, and tenth in closeness

Figure 2 presents the centrality analysis results, demonstrating sense of absence’s prominent but varied position across centrality measures. SoA achieved the highest expected influence (1.267), ranking 1 st among all nodes, and demonstrated strong performance in other centrality measures, ranking 2nd in betweenness centrality, 4th in strength centrality, and 10th in closeness centrality. The highest expected influence score indicates that SoA occupied the most influential structural position in the network, suggesting that it was strongly connected to other variables across domains. SoA’s strong betweenness centrality (2nd highest) indicates that it frequently lay on shortest paths between other nodes, consistent with a connector role in the network. The moderate strength centrality ranking indicates SoA has substantial but not maximal direct connectivity, while the lower closeness ranking suggests it may not be optimally positioned for rapid information transmission to all network nodes.

Most importantly, SoA’s expected influence (1.267) was statistically significantly higher than the network mean (bootstrap 95% CI: [0.338, 0.610], *p* < 0.05), representing 1.59 standard deviations above the mean and a 7.8% advantage over the second-ranked node (problematic social networks use: 1.175). Expected influence is particularly relevant for identifying structurally prominent nodes because it summarizes the signed strength of a node’s direct associations, making SoA’s dominance on this measure especially informative for understanding its role in the network.

### Empirical community structure and conventional bridge centrality

Before estimating bridge centrality, bootstrap exploratory graph analysis was used to identify the empirical community structure of the network. A three-community solution was recovered in all 5,000 bootstrap samples. Community 1 contained Sense of Absence, parent–adolescent communication, attachment avoidance, and attachment anxiety. Community 2 contained alcohol use, drug use, problem gambling, problematic pornography use, and compulsive sexual behavior. Community 3 contained internet gaming disorder, problematic social-network use, smartphone addiction, and shopping addiction. Structural consistency was 0.998 for Community 1, 1.000 for Community 2, and 0.765 for Community 3, and the corresponding average item-stability coefficients were 1.000, 1.000, and 0.941. Sense of Absence showed an item-stability coefficient of 1.00 and was assigned to the same community as parent–adolescent communication and attachment avoidance in 100% of the bootstrap samples and as attachment anxiety in 99.8% of the samples.

Table 2 presents the conventional bridge-centrality results based on these three empirically derived communities. Because the communities differed in size, normalized values were used for comparison. Sense of Absence had a normalized one-step bridge expected-influence value of 0.0297, ranking fourth among all 13 nodes and first within Community 1. The three nodes with higher one-step bridge expected influence were internet gaming disorder (0.0418), problem gambling (0.0396), and shopping addiction (0.0352).

**Table 2:** Conventional bridge-centrality indices across empirically derived communities

	One-step bridge expected influence			Two-step bridge expected influence			Bridge strength		
	Value	Overall rank	Community rank	Value	Overall rank	Community rank	Value	Overall rank	Community rank
**Relational and Sense of Absence**									
Sense of Absence	**0.0297**	**4**	**1**	**0.00508**	**3**	**1**	**0.0297**	**9**	**3**
Parent–Adolescent Communication	0.0229	8	2	0.00426	6	2	0.0385	7	2
Attachment Anxiety	0.0127	9	3	0.00311	10	3	0.0515	5	1
Attachment Avoidance	−0.0070	13	4	0.00010	13	4	0.0070	12	4
**Substance**,** gambling**,** and sexual behaviors**									
Problem Gambling	0.0396	2	1	0.00421	7	2	0.0950	1	1
Problematic Pornography Use	0.0271	5	2	0.00400	8	3	0.0551	4	2
Alcohol	0.0268	6	3	0.00524	2	1	0.0268	10	3
Compulsive Sexual Behavior	0.0068	10	4	0.00375	9	4	0.0203	11	4
Drugs	0.0000	12	5	0.00113	12	5	0.0000	13	5
**Digital and shopping behaviors**									
Internet Gaming Disorder	0.0418	1	1	0.00668	1	1	0.0621	3	2
Shopping Addiction	0.0352	3	2	0.00504	4	2	0.0756	2	1
Problematic Social-Network Use	0.0235	7	3	0.00495	5	3	0.0375	8	4
Smartphone Addiction	0.0028	11	4	0.00171	11	4	0.0454	6	3

Bolded values are significant at* p* < .05

Communities were identified using bootstrap exploratory graph analysis with 5,000 nonparametric resamples

Values are normalized to account for differences in community size

Bridge expected influence retains the signs of cross-community edges, whereas bridge strength sums their absolute values

Overall rank refers to rank among all 13 nodes; community rank refers to rank within the node’s empirically assigned community

A similar pattern emerged for two-step bridge expected influence. Sense of Absence had a normalized value of 0.00508, ranking third overall and first within Community 1. Internet gaming disorder showed the highest two-step bridge expected influence (0.00668), followed by alcohol use (0.00524). Thus, among the variables comprising the relational community, Sense of Absence showed the strongest signed direct and indirect connectivity with nodes in the other empirically identified communities.

**Fig. 3:**
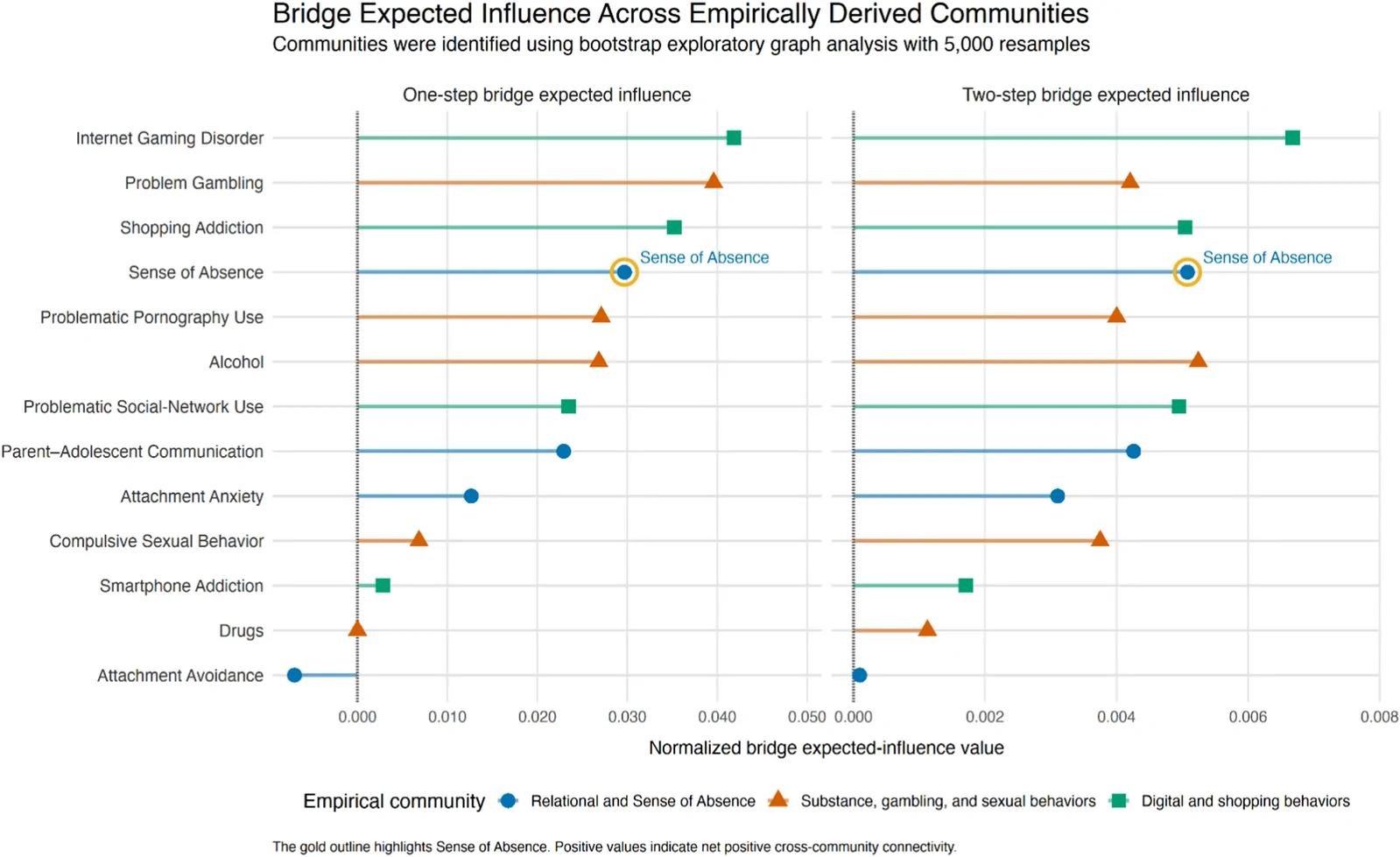
Bridge expected influence across empirically derived network communities. Communities were identified using bootstrap exploratory graph analysis with 5,000 nonparametric resamples. One-step bridge expected influence represents each node’s signed direct connectivity with nodes outside its assigned community, whereas two-step bridge expected influence additionally incorporates indirect connectivity through adjacent nodes. Colors and shapes indicate empirical community membership. The gold outline highlights Sense of Absence

The findings were less pronounced for bridge strength, which sums the absolute values of cross-community associations without retaining their signs. Sense of Absence had a normalized bridge-strength value of 0.0297, ranking ninth overall and third within Community 1. The highest bridge-strength values were observed for problem gambling (0.0950), shopping addiction (0.0756), and internet gaming disorder (0.0621). Figure 3 presents the normalized one-step and two-step bridge expected-influence values across the three communities. Overall, the results identify Sense of Absence as the leading signed cross-community connector within the relational community and as one of the more prominent bridge nodes in the network, although it was not the highest-ranking node across all bridge indices.

### Network stability and reliability

Network stability analysis was conducted using case-dropping bootstrap procedures to assess the robustness of centrality findings across different subsamples (Figure S1). The analysis evaluated the correlation stability coefficient (CS-coefficient), which indicates the maximum proportion of cases that can be dropped while maintaining a correlation of 0.7 with the original centrality values in at least 95% of bootstrap samples.

Results demonstrated excellent stability for the key centrality measures. Expected influence and strength centrality both achieved CS-coefficients of 0.75 (the highest level tested), indicating robust stability even when 75% of cases were dropped. Closeness centrality demonstrated good stability with a CS-coefficient of 0.361, while betweenness centrality showed moderate stability (CS-coefficient = 0.206). These stability coefficients exceed recommended thresholds (CS > 0.25 for acceptable stability, CS > 0.5 for good stability), confirming the reliability of the centrality findings.

The high stability of expected influence (CS = 0.75) is particularly important given SoA’s dominance in this measure, as it confirms that SoA’s highest expected influence ranking is robust across different subsamples of the data. Similarly, the excellent stability of strength centrality supports the reliability of the overall centrality pattern observed in the network. The stability analysis validates that the identified centrality relationships represent consistent structural patterns rather than sample-specific artifacts, strengthening confidence in SoA’s role as a critical network hub with maximal potential for network-wide influence.

## Discussion

Adolescent vulnerability to addictive behaviors may be understood, in part, in relation to inner emptiness and relational disconnection, with disruptions in attachment and reward systems making substances and certain repetitive behaviors appealing substitutes for diminished social bonding and natural reward. Building on this view, the present study examined the role of Sense of Absence (SoA) within a network that included parent-adolescent communication, attachment avoidance, attachment anxiety, and a range of addictive behaviors. Previous findings [7] suggest that SoA reflects relational invisibility, existential detachment, and disrupted self-continuity – a state in which adolescents may feel present yet unseen and struggle to participate, connect, or give and receive love. The present findings extend this account by showing that SoA was closely embedded within the relational part of the network while also maintaining meaningful connections with addictive behaviors.

The community analysis grouped SoA with parent-adolescent communication, attachment avoidance, and attachment anxiety. This pattern suggests that SoA is not an isolated experience but is closely tied to the broader relational context in which adolescents seek recognition, support, and emotional connection. At the same time, SoA showed the strongest positive connections from this relational community to the other communities in the network. It ranked first within its community on both one-step and two-step bridge expected influence and was among the more prominent bridge nodes in the full network. These findings are consistent with the idea that SoA may represent a subjective experience through which relational difficulties and addictive engagement are meaningfully connected. However, SoA was not the strongest node on every bridge index, indicating that it should be understood as an important relational-side connector rather than the only or universally dominant bridge.

From an attachment perspective, this pattern may reflect the importance of reliable relationships for emotional regulation and self-soothing. Secure attachment relationships provide a foundation for expressing, understanding, and regulating distress, whereas insecure or inconsistent relationships may leave adolescents with fewer internal and interpersonal resources for managing distress [36, 37]. Developmental models of addiction similarly suggest that when reliable relational soothing is unavailable, substances or repetitive behaviors may serve as external substitutes for regulation and relief [29, 31]. Therefore, placing SoA alongside attachment anxiety, attachment avoidance, and parent–adolescent communication is theoretically meaningful. It suggests that feeling unseen, disconnected, or unable to participate may be intertwined with difficulties in relying on others, expressing needs, or receiving dependable emotional support.

Family systems theory offers a complementary interpretation. Parent–adolescent communication is a key relational process through which emotional experiences are recognized and regulated within the family [23, 24]. When communication is open and responsive, adolescents may be better able to name distress, seek support, and remain engaged in relationships. When communication is distant, critical, or unreliable, feelings of absence may go unspoken and become harder to regulate. The present findings are consistent with this view because SoA and parent–adolescent communication fell within the same community and both were linked to addictive behaviors. This does not establish a temporal sequence, but it suggests that communication, attachment, and the subjective experience of absence are part of the same relational system.

Social identity and belonging perspectives further help explain why addictive behaviors may be relevant in this context. Adolescents who feel unseen, socially disconnected, or uncertain about their place in meaningful relationships may be especially drawn to activities that offer temporary participation, stimulation, or a substitute sense of connection [4]. Compensatory models propose that internet and media use may become particularly attractive when offline relationships, belonging needs, or emotional needs are perceived as unsatisfying [37, 50]. The presence of internet gaming disorder, problematic social-network use, smartphone addiction, and shopping addiction within a separate empirical community suggests that these behaviors may share compensatory or reinforcing functions, though they should not be assumed to operate in exactly the same way.

The findings can also be understood through the lenses of self-medication and emotion regulation. When relational regulation is weak, inconsistent, or unavailable, substances and repetitive behaviors may serve as external regulators by reducing distress, narrowing attention, providing reward, or creating temporary feelings of engagement [17]. In this sense, SoA may reflect more than social isolation alone. It may capture the felt absence of reliable soothing, emotional vitality, meaningful participation, and internalized support. Addictive engagement may become appealing because it offers a temporary response to this state, whether through numbing, stimulation, escape, or simulated connection. Emotion dysregulation may be particularly important in this process, as it has been identified as a factor linking insecure attachment to both substance-related and behavioral addictions [38, 51].

Neurobiological accounts offer an additional level of interpretation. Attachment, separation distress, stress regulation, and reward processing involve overlapping dopaminergic, opioid, oxytocinergic, and stress-related systems [52]. Relational disconnection may therefore be associated not only with emotional distress but also with shifts in the relative appeal of immediate and predictable rewards. Addictive behaviors may become salient because they provide rapid relief, stimulation, or temporary engagement when relational and natural rewards feel less available. This account is consistent with the current network pattern, but the cross-sectional design does not permit conclusions about the direction or sequence of these associations.

A transdiagnostic perspective also helps interpret the network’s diversity. Substance-related and behavioral addictions may share broad processes such as reinforcement, craving, compensation, impaired control, and the development of habitual patterns [35, 53]. Within this broader system, SoA may represent one upstream experiential vulnerability rather than a complete explanation for all forms of addictive engagement. The prominence of internet gaming disorder, problem gambling, shopping addiction, and alcohol use in bridge positions supports this more differentiated interpretation. Relational disconnection may be associated with addictive behavior through several routes, and different behaviors may provide distinct forms of temporary regulation or compensation.

The findings therefore contribute to understanding SoA as a potentially clinically relevant marker and highlight parent-adolescent communication as a potentially protective relational factor [27, 28, 54, 55] during a developmental period marked by vulnerability to addictive behaviors [56]. Emotional disconnection, attachment orientations, communication, and addictive behaviors appear to form an interconnected system linked to adolescents’ vulnerability or resilience. Using the SoAS alongside established measures of attachment, communication, loneliness, emotion regulation, and addictive behaviors may help identify adolescents whose distress involves relational invisibility and disengagement – experiences that broader measures of emptiness or loneliness may not fully capture. Clinical interpretation should nevertheless consider the broader relational and behavioral network rather than treating SoA as a single causal mechanism.

Interventions may benefit from strengthening reliable recognition, supportive communication, belonging, and meaningful participation [4]. Family-based work may offer opportunities to improve communication and interpersonal regulation, while group and school-based approaches may strengthen visibility, connection, and participation. Treatment planning may also draw on self-medication principles [17] by identifying the function a particular addictive behavior serves and by developing alternative relational and emotional sources of relief. School programs that cultivate belonging and visibility [57] may similarly help address the co-occurrence of relational absence and compensatory addictive behaviors during this critical developmental stage [35–38, 50–53].

Although the findings identify meaningful associations among SoA, relational tendencies, and addictive behaviors, several limitations should be noted. First, the current scale is broad and does not distinguish among specific relational sources of absence, such as parents, peers, or teachers. This may limit its ability to capture more nuanced interpersonal experiences. Future research would benefit from refining the measure to assess absence within distinct relational contexts. Second, the lack of a widely accepted definition of SoA presents challenges for its operationalization and interpretation, underscoring the need for further theoretical development and independent validation. Third, the cross-sectional design does not permit conclusions about temporal ordering, causality, mediation, or the direction of the observed associations. Although network analysis can identify conditional associations, empirical communities, and nodes that connect different parts of a network, it cannot determine whether relational difficulties precede SoA, whether SoA precedes addictive engagement, or whether these associations are reciprocal. Longitudinal research is therefore needed to examine how relational experiences, SoA, and addictive behaviors are associated across development.

The bridge findings should also be interpreted relative to the estimated network and the empirically identified community structure. SoA showed the highest one-step and two-step bridge expected influence within the relational community and ranked among the more prominent connector nodes in the overall network, but it was not the highest-ranking node across all bridge indices. Replication in independent samples is therefore needed to determine whether this structural pattern is stable across populations, developmental periods, and cultural contexts. Another limitation is the absence of clinical thresholds distinguishing normative from problematic levels of SoA, which currently limits its use for clinical classification or screening. Finally, the focus on Israeli adolescents aged thirteen to eighteen restricts generalizability. Subsequent research should examine possible differences by age, sex, cultural context, and developmental stage and should validate the SoAS in more diverse and clinical populations.

## Conclusion

The present study identifies SoA as a prominent relational-side connector within a network of attachment, parent-adolescent communication, and addictive behaviors among adolescents. SoA was empirically located within the relational community and showed the strongest signed cross-community connectivity among the variables in that community, although it was not the most prominent node across every bridge index. As a construct capturing inner emptiness, relational invisibility, diminished vitality, and difficulty participating meaningfully in life, SoA may help describe an experiential state that co-occurs with both relational difficulties and addictive engagement.

These findings suggest that addictive behaviors may, for some adolescents, be associated with attempts to obtain temporary relief, stimulation, escape, or a substitute sense of connection. However, the present results do not establish that SoA causes or mediates addictive behavior. Rather, they position SoA as one potentially important component of a broader relational and behavioral system. Further longitudinal and clinical research is needed before translating the construct into formal screening or intervention protocols. Nevertheless, the findings point to the potential value of attending not only to addictive symptoms but also to adolescents’ experiences of recognition, belonging, supportive communication, and meaningful participation in their social environments.

## Supplementary Information

Supplementary Material 1. Network centrality stability analysis. Case-dropping bootstrap stability curves showing the correlation between centrality measures computed on the full sample versus subsamples with varying proportions of cases removed. The y-axis represents the average correlation with the original sample, while the x-axis shows the percentage of cases remaining after random removal. Expected influence (teal) and strength (purple) demonstrate excellent stability with correlation stability coefficients (CS) of 0.75, maintaining high correlations even when 75% of cases are dropped. Closeness (green) shows good stability (CS = 0.361), while betweenness (red) displays moderate stability (CS = 0.206). Shaded areas represent 95% confidence intervals. All measures exceed recommended stability thresholds (CS > 0.25), confirming the robustness of centrality findings across different subsamples of the data.

## Data Availability

The analysis code associated with this study are available on the Open Science Framework at: https://osf.io/fvb5z/overview.

## Code availability

The analysis code associated with this study are available on the Open Science Framework at: https://osf.io/fvb5z/overview.
