# Supplementary material for: Filling the void: sense of absence as a comprehensive bridge between attachment, parent–adolescent communication, and addictive behaviors among adolescents
Source: Child Adolesc Psychiatry Ment Health. 2026 Aug 5;20:152. doi: 10.1186/s13034-026-01150-0 (PMC13729574; doi:10.1186/s13034-026-01150-0)
Supplement: Supplementary file 1 [file 13034_2026_1150_MOESM1_ESM.docx]

**Supplementary Figure 1**

**Network Centrality Stability Analysis**. Case-dropping bootstrap stability curves showing the correlation between centrality measures computed on the full sample versus subsamples with varying proportions of cases removed. The y-axis represents the average correlation with the original sample, while the x-axis shows the percentage of cases remaining after random removal. Expected influence (teal) and strength (purple) demonstrate excellent stability with correlation stability coefficients (CS) of 0.75, maintaining high correlations even when 75% of cases are dropped. Closeness (green) shows good stability (CS = 0.361), while betweenness (red) displays moderate stability (CS = 0.206). Shaded areas represent 95% confidence intervals. All measures exceed recommended stability thresholds (CS > 0.25), confirming the robustness of centrality findings across different subsamples of the data.
